# MicroRNA396 negatively regulates shoot regeneration in tomato

**DOI:** 10.1093/hr/uhad291

**Published:** 2024-01-02

**Authors:** Su-Jin Park, Ji-Sun Park, Jin Ho Yang, Ki-Beom Moon, Seung Yong Shin, Jae-Heung Jeon, Hyun-Soon Kim, Hyo-Jun Lee

**Affiliations:** Plant Systems Engineering Research Center, Korea Research Institute of Bioscience and Biotechnology, 125 Gwahak-ro, Daejeon 34141, Korea; Department of Biosystems and Bioengineering, KRIBB School of Biotechnology, University of Science and Technology, 125 Gwahak-ro, Daejeon 34113, Korea; Plant Systems Engineering Research Center, Korea Research Institute of Bioscience and Biotechnology, 125 Gwahak-ro, Daejeon 34141, Korea; Plant Systems Engineering Research Center, Korea Research Institute of Bioscience and Biotechnology, 125 Gwahak-ro, Daejeon 34141, Korea; Plant Systems Engineering Research Center, Korea Research Institute of Bioscience and Biotechnology, 125 Gwahak-ro, Daejeon 34141, Korea; Plant Systems Engineering Research Center, Korea Research Institute of Bioscience and Biotechnology, 125 Gwahak-ro, Daejeon 34141, Korea; Department of Functional Genomics, KRIBB School of Bioscience, University of Science and Technology, 125 Gwahak-ro, Daejeon 34113, Korea; Plant Systems Engineering Research Center, Korea Research Institute of Bioscience and Biotechnology, 125 Gwahak-ro, Daejeon 34141, Korea; Plant Systems Engineering Research Center, Korea Research Institute of Bioscience and Biotechnology, 125 Gwahak-ro, Daejeon 34141, Korea; Department of Biosystems and Bioengineering, KRIBB School of Biotechnology, University of Science and Technology, 125 Gwahak-ro, Daejeon 34113, Korea; Plant Systems Engineering Research Center, Korea Research Institute of Bioscience and Biotechnology, 125 Gwahak-ro, Daejeon 34141, Korea; Department of Functional Genomics, KRIBB School of Bioscience, University of Science and Technology, 125 Gwahak-ro, Daejeon 34113, Korea; Department of Biological Sciences, Sungkyunkwan University, 2066 Seobu-ro, Suwon 16419, Korea

## Abstract

Numerous studies have been dedicated to genetically engineering crops to enhance their yield and quality. One of the key requirements for generating genetically modified plants is the reprogramming of cell fate. However, the efficiency of shoot regeneration during this process is highly dependent on genotypes, and the underlying molecular mechanisms remain poorly understood. Here, we identified microRNA396 (miR396) as a negative regulator of shoot regeneration in tomato. By selecting two genotypes with contrasting shoot regeneration efficiencies and analyzing their transcriptome profiles, we found that miR396 and its target transcripts, which encode GROWTH-REGULATING FACTORs (GRFs), exhibit differential abundance between high- and low-efficiency genotypes. Suppression of miR396 functions significantly improved shoot regeneration rates along with increased expression of *GRF*s in transformed T0 explants, suggesting that miR396 is a key molecule involved in the determination of regeneration efficiency. Notably, we also showed that co-expression of a miR396 suppressor with the gene-editing tool can be employed to generate gene-edited plants in the genotype with a low capacity for shoot regeneration. Our findings show the critical role of miR396 as a molecular barrier to shoot regeneration in tomato and suggest that regeneration efficiency can be improved by blocking this single microRNA.

## Introduction

Modern plant biotechnology has enabled the engineering of target genes to confer useful traits, such as improved environmental adaptability, resistance to pathogens, high survival rates under stress conditions, and increased crop yield [1, 2]. In many cases, genetically engineered plants are generated by inducing shoot regeneration through reprogramming of transformed somatic cells [3]. For shoot regeneration, plant tissues are typically excised and incubated on media containing auxins and cytokinins, which induce cell fate transitions [4]. The auxin-to-cytokinin ratio is important for this process. Incubation of explants on a medium containing high levels of auxins, known as a callus-inducing medium (CIM), can trigger the formation of callus with root identity and pluripotency [5]. Subsequently, incubation of calli on a medium with a high cytokinin-to-auxin ratio, known as a shoot-inducing medium (SIM), changes in the identity of the callus cells from root to shoot, thus triggering the formation of the shoot meristem. Molecular events during these processes have been mainly studied in *Arabidopsis thaliana* (Arabidopsis). During the incubation on the CIM, AUXIN RESPONSE FACTOR transcription factors are activated by auxin, resulting in the up-regulation of root identity genes such as *LATERAL ORGAN BOUNDARIES DOMAIN*s, which promote callus formation [6]. Simultaneously, auxin activates *PLETHORA* and *CUP-SHAPED COTYLEDON 2* genes for acquisition of competence to shoot regeneration [4, 7]. When the calli are transferred to SIM, the high cytokinin-to-auxin ratio triggers the activation of type B ARABIDOPSIS RESPONSE REGULATOR (ARR) transcription factors leading to the up-regulation of *WUSCHEL* (*WUS*), a transcription factor that plays a key role in potentiating *de novo* shoot organogenesis [8–10]. However, limited studies have reported molecular mechanisms of the callus-mediated shoot regeneration process in crops. In tomato, it was shown that the expression of five representative genes (*SlARR1, Sl-CYCLIN3;1, Sl-SHOOT MERISTEMLESS, Sl-WOUND INDUCED DEDIFFERENTIATION 1,* and *SlWUS*) involved in shoot regeneration largely increases during tissue culture in cv. Micro-Tom, which exhibits a high regeneration capacity [11]. In cotton (*Gossypium hirsutum*), two homologs of Arabidopsis *WUS*, *GhWUS*s, have been identified and found to act as positive regulators for both callus and shoot formation [12]. In rice (*Oryza sativa*), a homolog of Arabidopsis *WUSCHEL-RELATED HOMEOBOX* (*WOX5*), *OsWOX5,* has been found to promote both callus pluripotency and shoot regeneration [13]. These reports suggest that molecular mechanisms of shoot regeneration are widely conserved in crops.

While the key genes involved in shoot regeneration were identified, molecular events determining low- and high-efficiency genotypes remain elusive in most crop species. For example, tomato (*Solanum lycopersicum*) cultivar (cv.) Riograndea shows ~65% of the shoot regeneration rate, whereas cv. Feston only shows 8% [14]. In addition, Chinese cabbage (*Brassica rapa*) cv. Beijing New No. 3 shows ~62% of the shoot regeneration rate, whereas cv. FT shows 0% [15]. Low shoot regeneration capacity can pose a bottleneck in developing commercially novel varieties through genetic engineering, as it requires generating a large number of transgenic plants to screen out those with unintended changes such as multiple copies of the T-DNA, insertion of vector backbone fragments, and random mutations [16, 17].

To overcome low regeneration capacity in several crop genotypes, ectopic expression of genes functioning as an activator of shoot regeneration has been applied. Ectopic coexpression of the maize (*Zea mays*) *BABY BOOM (BBM)* and *WUS2* genes by *Agrobacterium tumefaciens*-mediated transformation elevated transformation efficiency in monocot plants such as rice, sorghum (*Sorghum bicolor*), and sugarcane (*Saccharum officinarum*) [18]. Recently, ectopic expression of *GROWTH-REGULATING FACTOR 4* (*GRF4*) and its cofactor *GRF-INTERACTING FACTOR 1* (*GIF1*) was shown to improve shoot regeneration efficiency of wheat (*Triticum aestivum*) in transformed T0 explants [19]. The *GRF4-GIF1*-transformed calli developed shoots even in the absence of exogenous cytokinins, showing that this method can be used to discriminate transgenic and nontransgenic calli. Indeed, coexpression of GRF4-GIF1 chimera with CLUSTERED REGULARLY INTERSPACED SHORT PALINDROMIC REPEATS (CRISPR)/CRISPR-ASSOCIATED PROTEIN 9 (Cas9) gene editing tools increased the rate of recovering gene-edited plants from the transformed calli. In addition, overexpression of Arabidopsis *GRF5* has been shown to be effective in improving shoot regeneration in sugar beet (*Beta vulgaris*), canola (*Brassica napus*), soybean (*Glycine max*), and sunflower (*Helianthus annuus*) [20], suggesting that GRF family members are involved in shoot regeneration. MicroRNA396 (miR396) is a known upstream regulator of the GRF family genes. However, miR396 functions have mainly been studied in relation to plant growth. MiR396 controls organ size and biomass yield in Arabidopsis, tomato, and switchgrass (*Panicum virgatum*) by post-transcriptionally repressing multiple *GRF* transcripts [21–23]. In rice, blocking miR396 by overexpressing target mimicry increases grain yield through the upregulation of *OsGRF6* and auxin biosynthesis [24]. In Medicago, miR396 inactivation increases the expression of *MtGRF*s, resulting in improved root biomass and mycorrhizal associations [25]. These results indicate that the function of miR396 is widely conserved in various crop species. Notably, while a previous study showed the involvement of miR396 in auxin-mediated somatic embryogenesis [26], its potential role in shoot regeneration remains to be elucidated.

In this study, we found that miR396 acts as a repressor of shoot regeneration and plays a role in determining the genotype-dependent differences in regeneration capacity in tomato. Time-course transcriptome analysis with small RNA-sequencing revealed that abundance of miR396 and its target *GRF* transcripts is highly different in two genotypes showing low and high regeneration capacity. Suppressing miR396 functions significantly improved rate of shoot regeneration in the low-efficiency genotype, suggesting that miR396 is a negative regulator of *de novo* shoot organogenesis. Furthermore, we showed that a miR396 suppressor can be used to generate gene-edited plants in low-efficiency genotype. Our study revealed that miR396 not only acts as a molecular barrier for shoot regeneration in tomato but also can be used to improve regeneration efficiency by regulating its functions.

## Results

### Transcriptome analysis of two genotypes showing different shoot regeneration capacity

**Figure 1:**
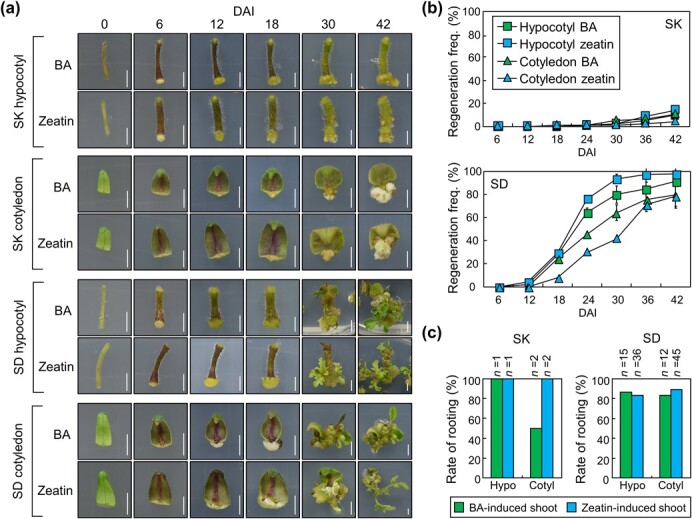
Regeneration efficiency of two different tomato genotypes. For (**b**), whiskers indicate standard deviation of the mean (sd). **a** Representative image of explants during shoot regeneration. Hypocotyl and cotyledon explants from tomato seedlings grown for 6 days were incubated on SIM. SK, Sweet King; SD, Super Doterang. Scale bars, 0.5 cm. **b** Shoot regeneration frequency in hypocotyl and cotyledon explants. The explants were incubated on SIM containing either BA or zeatin for the specified durations. The regeneration frequency was determined by dividing the number of explants producing regenerated shoots by the total number of explants (means ± sd; *n* = 3 biological replicates). Approximately 80 explants were used per replicate. DAI, days after incubation. **c** Rooting rate of regenerated shoots. The regenerated shoots were excised from the callus at 42 DAI and grown on MS-agar medium for 2 weeks. Hypo, hypocotyl; Cotyl; cotyledon.

To identify key molecules that determine shoot regeneration capacity in tomato, we selected two genotypes, Sweet King (SK) and Super Doterang (SD), which exhibit different levels of shoot regeneration capacity. Incubation of hypocotyl and cotyledon explants of these genotypes on SIM induced *de novo* shoot organogenesis, but regeneration efficiency was highly different (Fig. 1a and b). Regeneration frequency was up to 14% at 42 days after incubation (DAI) in SK, while that was up to 97% in SD. Hypocotyl explants showed a slightly higher rate of shoot regeneration than cotyledon explants in SD, but both tissues exhibited extremely low regeneration efficiency in SK (Fig. 1b). We used two types of cytokinins, benzyladenine (BA) and zeatin, but both cytokinins did not show significant differences in final regeneration frequency. In detail, shoot regeneration was first observed in SD hypocotyl at 9 DAI and in SD cotyledon at 15 DAI, while shoot regeneration was not observed in SK at those time points. Callus formation was first observed at 6 DAI in both SD and SK, regardless of tissue types. This suggests that shoot regeneration, rather than callus formation, is affected by genotypes.

Rooting rate of the regenerated shoots was more than 80% in both SK and SD except for shoots regenerated from SK cotyledon explants on BA-containing SIM (Fig. 1c). A low rooting rate in this condition might be because only a small number of shoots were used for the analysis (*n* = 2) due to extremely low regeneration efficiency in SK. These results suggest that signaling pathways for cytokinin-induced shoot regeneration are genetically blocked in SK.

**Figure 2:**
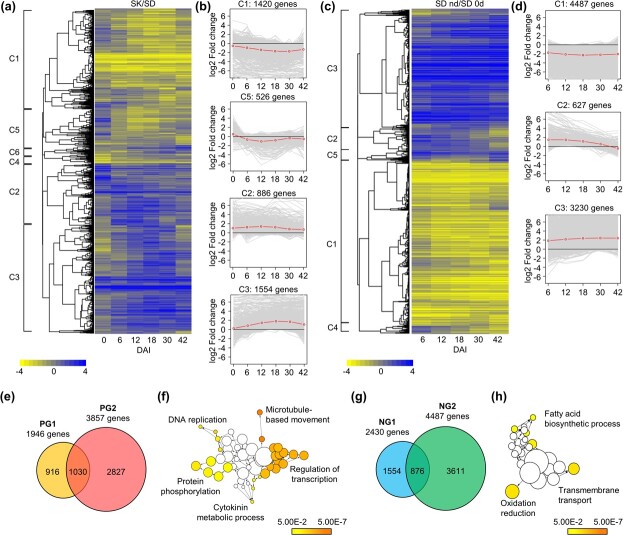
Transcriptome analysis on SD and SK explants during shoot regeneration. **a, b** Clustering analysis on DEGs between SK and SD explants during regeneration. Expression levels of DEGs are represented as a heatmap (**a**). Scale bar, log2 fold change. The clusters that contain genes exhibiting reduced expression (C1 and C5) and those exhibiting increased expression (C2 and C3) in SK are displayed (**b**). **c, d** Clustering analysis on DEGs during shoot regeneration in SD. Expression levels of DEGs are represented as a heatmap (**c**). Scale bar, log2 fold change. The clusters that contain genes exhibiting reduced expression (C1) and those exhibiting increased expression (C2 and C3) during SIM incubation are displayed (**d**). d, DAI. **e** Venn diagram showing putative positive regulators of shoot regeneration. Genes belonging to clusters C1 or C5 in (**b**) were designated as PG1, while those belonging to clusters C2 or C3 in (**d**) were designated as PG2. **f** GO analysis of 1030 intersection genes found in (**e**). Colored nodes indicate significantly over-represented GO terms (Benjamini–Hochberg-corrected *P* < 0.01). Scale bar, *P*-values. **g** Venn diagram showing putative negative regulators of shoot regeneration. Genes belonging to clusters C2 or C3 in (**b**) were designated as NG1 and those belonging to cluster C1 in (**d**) were designated as NG2. **h** GO analysis of 876 intersection genes found in (**g**). Colored nodes indicate significantly over-represented GO terms (Benjamini–Hochberg-corrected *P* < 0.01). Scale bar, *P*-values.

We next performed time-course transcriptome sequencing of these two genotypes during shoot regeneration to find clues on the difference of regeneration capacity at the molecular level. Firstly, we analyzed differentially expressed genes (DEGs) between SK and SD at each time point of SIM incubation. Clustering DEGs based on expression patterns resulted in six clusters (Fig. 2a and Supplementary Table S1). Among the clusters, cluster 1 (C1) and C5 contained genes showing lower average expression levels in SK than those in SD during the shoot regeneration (Fig. 2b). On the contrary, genes belonging to C2 and C3 showed higher average expression levels in SK than those in SD. Because SK exhibited lower regeneration capacity than SD (Fig. 1a and b), we hypothesized that expression levels of positive regulators for shoot regeneration in SK are lower than those in SD, and negative regulators show opposite patterns. We thus grouped the genes in C1 and C5 into a putative positive regulator group (PG1) and those in C2 and C3 into a putative negative regulator group (NG1) of shoot regeneration.

Next, we analyzed DEGs at each time point during SIM incubation in the high-efficiency genotype SD, compared to 0 DAI. Again, we clustered DEGs based on the expression patterns and found that there are five clusters (Fig. 2c and Supplementary Table S2). Among the clusters, genes belonging to C1 showed low average expression during the regeneration, while those belonging to C2 and C3 showed high average expression (Fig. 2d). Because SD exhibited high regeneration capacity, we hypothesized that expression levels of positive regulators for shoot regeneration increase during the SIM incubation, while those of negative regulators decrease. Therefore, we grouped genes in C2 and C3 into a putative positive regulator group (PG2) and those in C1 into a putative negative regulator group (NG2) of shoot regeneration.

We tried to identify positive and negative regulators of shoot regeneration by combining two types of DEG analyses described above. The comparison of PG1 and PG2 revealed that 1030 genes were common to both groups and were designated as a PG3 (Fig. 2e and Supplementary Table S3). Gene ontology (GO) analysis of PG3 genes showed that roughly five types of GO terms are over-represented, with notably low *P*-values observed for GO terms related to regulation of transcription and microtubule-based movement (Fig. 2f and Supplementary Table S4). Notably, one of the over-represented GO terms is cytokinin metabolic process, which includes three cytokinin oxidase genes (Solyc04g080820, Solyc12g008900, and Solyc04g016430). As cytokinin oxidase induces the degradation of cytokinins [27], it is possible that maintaining cytokinin homeostasis may be required for efficient shoot regeneration.

Comparing NG1 and NG2 revealed that 876 genes were common to both groups (NG3; Fig. 2g and Supplementary Table S5). GO analysis of NG3 genes showed that fatty acid biosynthetic process, oxidation–reduction, and transmembrane transport-related terms are over-represented, but they showed similar *P*-values (Fig. 2h and Supplementary Table S4).

### The GRF-miR396 module determines genotype-dependent shoot regeneration capacity in tomato

To identify genes responsible for genotype-dependent difference in shoot regeneration, we initially analyzed the expression of regeneration-related genes whose function has been reported previously in Arabidopsis [4] (Supplementary Table S6). Among those genes, we specifically focused on those belonging to PG3. There are 10 PG3 genes, with most known to be related to auxin signaling [4]. Therefore, we examined whether auxin treatment could enhance the efficiency of shoot regeneration in SK. However, auxin only marginally increased shoot regeneration efficiency (Supplementary Fig. S1), suggesting that auxin is not the primary signal limiting shoot regeneration capacity in SK.

**Figure 3:**
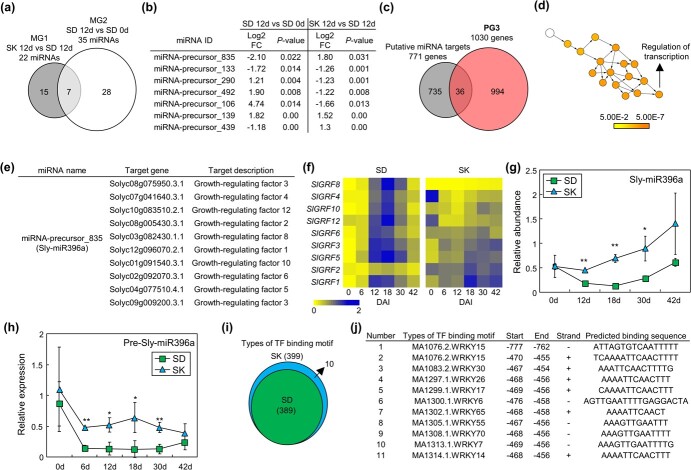
Comparative analysis of RNA-seq and small RNA-seq on SK and SD explants during regeneration. For (**g**) and (**h**), whiskers indicate sd. Statistical significance was analyzed using Student’s *t*-test (^*^P < 0.05; ^**^P < 0.01; means ± sd; *n* = 3 biological replicates). **a** Venn diagram showing the differentially abundant miRNAs during shoot regeneration. **b** List of seven intersection miRNAs found in (**a**). FC, fold change. **c** Venn diagram showing putative miRNA-target positive regulators of shoot regeneration. Genes encoding target transcripts of the seven miRNAs in (**b**) and those identified in Fig. 2e were used for the analysis. **d** GO analysis of 36 intersection genes found in (**c**). Colored nodes indicate significantly over-represented GO terms (Benjamini–Hochberg-corrected *P* < 0.01). Scale bar, *P*-values. **e** List of genes encoding miRNA target transcripts whose GO terms belong to ‘regulation of transcription’ analyzed in (**d**). **f** Heatmap showing expression of the nine different *SIGRF*s in SK and SD explants during shoot regeneration. Average values of relative expression were displayed (*n* = 4–5). **g** Abundance of Sly-miR396a in SK and SD explants during shoot regeneration. Sly-miR396a abundance was analyzed by the stem-loop qRT-PCR method. **h** Abundance of pre-Sly-miR396a in SK and SD explants during shoot regeneration. Pre-Sly-miR396a abundance was determined by the qRT-PCR method. **i** Venn diagram showing types of transcription factor (TF) binding motif in promoter sequences of miR396a. TF binding motif was analyzed in JASPAR. **j** List of unique TF binding motif in SK. Types of TF binding motif found exclusively in SK (**i**) were displayed, along with their binding locations in the promoter sequences.

We thus tried to identify primary genes determining regeneration capacity in different genotypes. However, there are too many genes in PG3 to further examine their functions. Therefore, we analyzed differentially abundant miRNAs during shoot regeneration by small RNA-sequencing to find upstream regulators of these DEGs. We chose to focus on the 12 DAI time point, as this is when explants in the high-efficiency genotype SD began to show signs of shoot regeneration, while the low-efficiency genotype SK did not (Fig. 1b). By comparing SK and SD, we identified 22 miRNAs showing different abundance between these genotypes and classified them into a new miRNA group, MG1 (Fig. 3a and Supplementary Table S7). In addition, we analyzed the abundance of miRNAs before and after inducing shoot regeneration in SD, and identified 35 differentially abundant miRNAs, which were grouped as MG2 (Fig. 3a and Supplementary Table S8). A comparative analysis of MG1 and MG2 showed that seven miRNAs are present in both groups (Fig. 3b). Using sequence-based prediction, we analyzed 771 genes as potential targets of the seven miRNAs (Fig. 3c and Supplementary Table S9). To find positive or negative regulators responsible for genotype-dependent different efficiency of shoot regeneration regulated by miRNAs, we performed a comparative analysis of small RNA-sequencing and transcriptome data. The results revealed 36 and 21 genes that belong to PG3 and NG3, respectively (Fig. 3c and Supplementary Fig. S2; Supplementary Tables S10 and S11). To further narrow down genes involved in shoot regeneration, we performed a GO analysis of the miRNA-target PG3 and NG3 genes. Although no over-represented GO terms were identified among the 21 NG3 genes, a significant over-representation of the GO term related to regulation of transcription was observed in the 36 PG3 genes (Fig. 3d). Notably, all 10 genes identified in this GO term encode GRFs (Fig. 3e). A miRNA targeting these *GRF*s was miRNA-precursor_835, which is known as Sly-miR396a in tomato [22].

Recent studies have reported that *GRF*s improve shoot regeneration efficiency in many plant species [19, 20]. Thus, we hypothesized that different abundance of *GRF* transcripts and Sly-miR396a is related to different regeneration capacity in SK and SD. The time-course analysis of *SlGRF*s expression showed that the average expression of *SlGRF*s was highly elevated at 12–18 DAI in SD, but remained largely unaltered in SK during the SIM incubation (Fig. 3f). Contrary to the *SlGRF*s expression, abundance of Sly-miR396a in SK was significantly higher than that in SD during the SIM incubation (Fig. 3g). Together with the previous reports that miR396 negatively regulates *GRF* transcripts [22], these data suggest that abundance of Sly-miR396a-*SlGRF* module controls shoot regeneration capacity in tomato genotypes.

We next analyzed the transcript levels of precursor Sly-miR396a (pre-Sly-miR396a) during shoot regeneration. Similar to the mature Sly-miR396a levels, the abundance of pre-Sly-miR396a in SK was significantly higher than that in SD (Fig. 3h). These results suggest that the transcriptional regulation of Sly-miR396a plays a major role in determining genotype-dependent differences in Sly-miR396a levels. To find the genetic variance related to the *Sly-MIR396A* transcription, we analyzed transcription factor binding motifs in the promoter sequence of *Sly-MIR396A* using the JASPAR database [28] (Supplementary Fig. S3a). The promoter sequence in SD contains 4485 transcription factor binding motifs, comprising 389 distinct types (Fig. 3i and Supplementary Table S12). In contrast, the promoter sequence in SK contains 4307 binding motifs, representing 399 distinct types (Fig. 3i and Supplementary Table S13). Notably, unique transcription factor binding motifs were found exclusively in SK, all of which are motifs for WRKY transcription factors (Fig. 3i and j). Notably, most of these unique binding motifs are located in a similar region, except a #1 WRKY15 binding motif (Supplementary Fig. S3b). These data suggest that DNA sequence polymorphisms resulting in additional binding motifs may induce different transcriptional regulation in SK.

**Figure 4:**
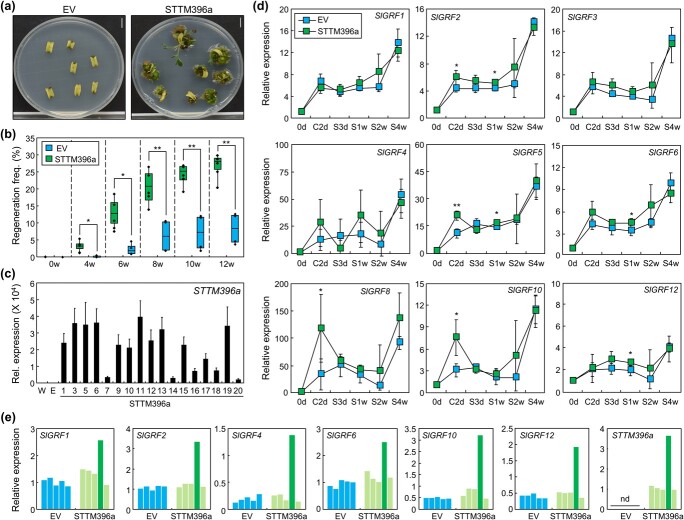
Suppressing Sly-miR396a improves shoot regeneration efficiency in tomato explants. For (**c**) and (**d**), whiskers indicate sd. **a** Representative images of transformed T0 explants. An empty vector (EV) or a pH2GW7-STTM396a vector (STTM396a) was transformed into wild-type SK using *Agrobacterium*. Explants at 12 weeks after incubation were displayed. Scale bars, 1 cm. **b** Effects of *STTM396a* overexpression on shoot regeneration. Shoot regeneration frequency of the transformed T0 explants was determined by dividing the number of explants producing regenerated shoots by the total number of explants. Statistical significance was analyzed using Student’s *t*-test (^*^P < 0.05; ^**^P < 0.01; *n* = 4–5 biological replicates). Each experiment utilized 70–100 explants. w, weeks after incubation. **c** Expression of *STTM396a* in regenerated shoots. Representative 17 regenerated shoots were analyzed (means ± sd; *n* = 3 technical replicates). W, wild type; E, empty vector. **d** Expression of the nine different *SlGRF*s in transformed T0 explants during shoot regeneration. Statistical significance was analyzed using Student’s *t*-test (^*^P < 0.05; ^**^P < 0.01; mean ± sd; *n* = 4–5 biological replicates). C, cocultivation medium; S, SIM. **e** Expression of *SlGRF*s and *STTM396a* in individual replicates at S2w. Relative expression of each replicate at S2w in (**d**) was redisplayed. nd, not detected.

To verify the role of Sly-miR396a in regulating shoot regeneration capacity, we employed the short tandem target-mimic (STTM) system, which functions as a target mimicry to suppress the activity of miRNAs [29]. The *STTM396a*-overexpressing vector (pH2GW7-STTM396a) was transformed into the low-efficiency SK explants using the *Agrobacterium*-mediated transformation method and regeneration efficiency at T0 stage was analyzed. The empty vector (EV) was used as a control. The transformation of the *STTM396a*-overexpressing vector significantly elevated shoot regeneration efficiency from 4 to 12 weeks after SIM incubation, resulting in a final average regeneration efficiency of 27%, which was significantly higher than that observed in EV-transformed SK with an efficiency of 7% (Fig. 4a and b). Most of the regenerated shoots analyzed from the pH2GW7-STTM396a-transformed SK explants were found to be expressing *STTM396a* transcripts (85%; Fig. 4c), implying that the transformation with *STTM396a* significantly induced high regeneration efficiency. Note that rooting is not affected by *STTM396a* overexpression (Supplementary Fig. S4). Because *GRF*s are known targets of miR396, we analyzed whether suppression of Sly-miR396a function by introducing *STTM396a* affects *GRF*s expression. During the process of shoot regeneration, time-course analysis of transformed T0 SK explants revealed that the expression levels of *SlGRF2*, *SlGRF5*, *SlGRF6*, *SlGRF8*, *SlGRF10*, and *SlGRF12* were significantly higher in pH2GW7-STTM396a-transformed explants compared to EV-transformed explants at several time points (Fig. 4d). This observation was made despite the heterogeneous nature of T0 explants, which may contain both transformed and nontransformed cells. Because of the high variation among replicates, we compared the expression of *SlGRF*s in each replicate with *STTM396a* (Fig. 4e and Supplementary Fig. S5). Among the time points, S2w showed particularly high variation in the expression of *SlGRF*s. Analysis of each replicate revealed that the fourth replicate exhibited exclusively high expression of all *SlGRF*s, consistent with the high expression of *STTM396a* in this replicate (Fig. 4e). These data suggest that the high expression of *STTM396a* leads to elevated expression of *SlGRF*s, particularly at S2w.

**Figure 5:**
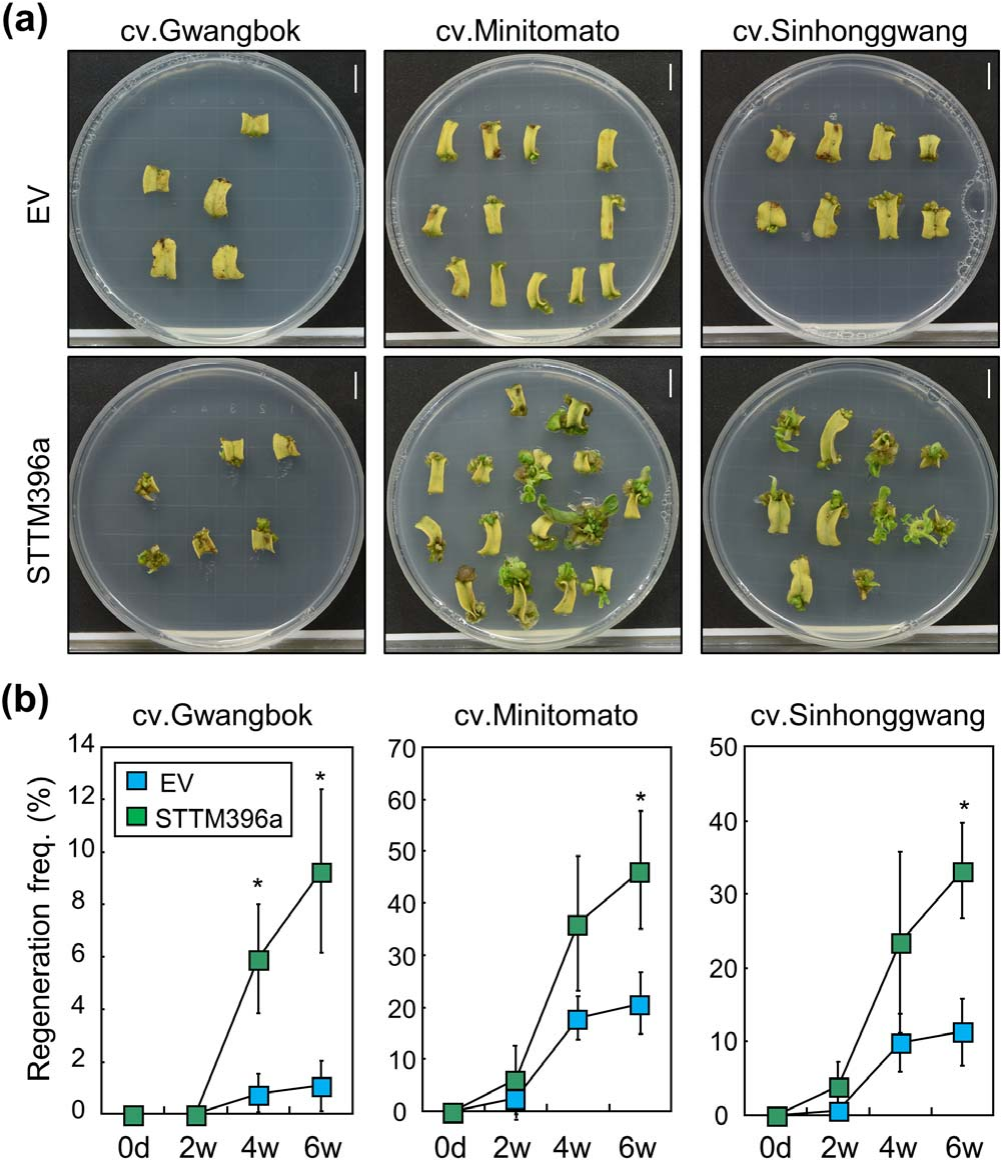
Effects of *STTM396a* overexpression on shoot regeneration in other genotypes. **a** Photographs of shoot regeneration from T0 transformed leaf explants into three tomato genotypes. An EV or a STTM396a was transformed into three different genotypes, Gwangbok, Sinhonggwang, and Minitomato. Explants at 6 weeks after incubation were displayed. Scale bars, 1 cm. **b** Regeneration frequency after STTM396a transformation in three tomato genotypes. Shoot regeneration frequency of the transformed T0 explants was determined by dividing the number of explants producing regenerated shoots by the total number of explants. Statistical significance was analyzed using Student’s *t*-test (^*^P < 0.05; *n* = 3 biological replicates). Each experiment utilized 50–120 explants.

To verify the effect of *STTM396a* in other tomato genotypes, we transformed the *STTM396a*-overexpressing vector into three different genotypes: Gwangbok, Minitomato, and Sinhonggwang. *STTM396a* overexpression increased regeneration efficiency in all three genotypes (Fig. 5a and b). The average regeneration efficiency in *STTM396a*-overexpressed explants was ~2.36–4.29-fold higher than that in EV-transformed explants (Fig. 5b). These results indicate that the low shoot regeneration capacity in tomato can be overcome by introducing *STTM396a*, which suppresses the function of Sly-miR396a and increases the expression of multiple *SlGRF*s.

### Generation of gene-edited plants in low-efficiency genotype by introducing *STTM396a*

**Figure 6:**
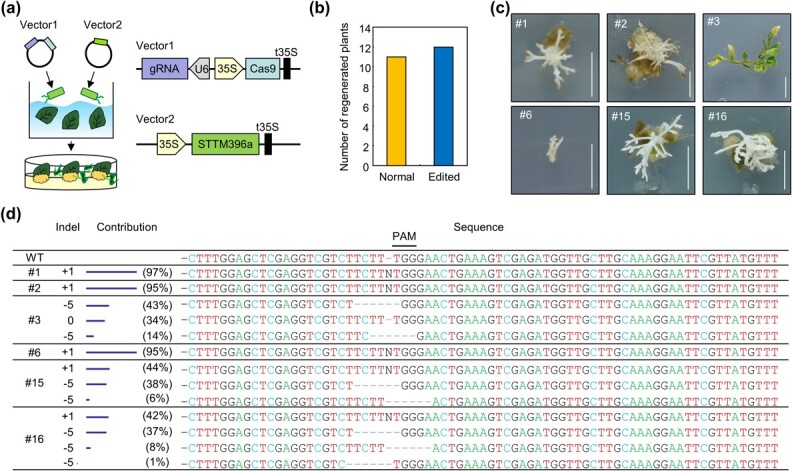
Generating gene-edited plants by cotransforming vectors expressing *Cas9-gRNA* and *STTM396a*. **a** Experimental scheme showing *Agrobacterium*-mediated cotransformation of vectors expressing *Cas9-gRNA* and *STTM396a*. **b** Number of regenerated plants after cotransformation of vector 1 and vector 2. The Cas9-gRNA vector for editing *SlPDS* and the pH2GW7-STTM396a vector for overexpression of *STTM396a* were cotransformed into wild-type SK explants. Number of regenerated shoots was displayed. Normal, nonedited plants; edited, *SlPDS*-edited plants. Editing of *SlPDS* was identified by Sanger sequencing. **c** Phenotype of *SlPDS*-edited shoots harboring both *Cas9* and *STTM396a*. Scale bars, 1 cm. **d** Sequencing of target regions in transgenic shoots. Regenerated transgenic shoots in (**c**) were used for the analysis. Target regions for CRISPR-Cas9-mediated gene editing were amplified by PCR and were subjected to Sanger sequencing. The ICE analysis represents the indel pattern in each target region.

Based on our data that *STTM396a* elevates shoot regeneration, we anticipated that *STTM396a* can be used for generation of gene-edited plants in SK. Thus, we cotransformed Cas9-gRNA vector targeting a gene encoding phytoene desaturase (SlPDS) and pH2GW7-STTM396a vector overexpressing *STTM396a* in SK explants (Fig. 6a). We obtained 23 regenerated shoots, among which 12 shoots possessed indels in the *SlPDS* locus (Fig. 6b). Most of the *SlPDS*-edited plants showed albino or chimeric phenotype (Fig. 6c), as reported previously [30]. Inference of CRISPR Edits (ICE) analysis showed that there was a nucleotide insertion or deletion in the upstream regions of the protospacer adjacent motif site (Fig. 6d), which is a typical feature observed in CRISPR/Cas9-edited plants [31]. These results indicate that suppressing Sly-miR396a function by introducing *STTM396a* can be used to generate gene-edited plants in low-efficiency tomato genotype.

## Discussion

In this study, we performed time-course transcriptome analysis of two genotypes showing different shoot regeneration capacity during shoot regeneration. By comparing the transcriptome sequencing and small RNA-sequencing data, we identified that the abundance of miR396a plays a key role in determining the efficiency of shoot regeneration. We found that Sly-miR396a and its target transcripts encoding *SlGRFs* show different abundance between high-efficiency and low-efficiency genotypes. In addition, gene-edited plants can be generated by cotransformation of gene editing tools and the *STTM396a* overexpression vector in low-efficiency genotype. Previous studies have shown that overexpression of a specific *GRF* improves plant regeneration. In wheat, overexpression of *TaGRF4-TaGIF1* chimera enhanced shoot regeneration efficiency, but *TaGRF1-TaGIF1* chimera was less effective [19]. In sugar beet, canola, soybean, and sunflower, *AtGRF5* was used to elevate regeneration efficiency [20]. However, there are numerous *GRF* family genes in plants [32, 33]; hence, selecting the effective *GRF* gene requires individual studies for each plant species. Conversely, miR396a, an upstream regulator of *GRF*s, is perfectly identical across multiple plant species, including crops (Supplementary Table S14). Moreover, the introduction of a miR396a suppressor is relatively easy due to the short length of miR396a (21 nucleotides). Therefore, the enhancement of shoot regeneration efficiency through ectopic expression of *STTM396a*, as observed in our study, has the potential to be applied to other crops.

Although there are numerous genes known to improve shoot regeneration efficiency, it is challenging to employ these genes for assisting in the generation of genetically engineered plants due to the side effects typically caused by the overexpression of shoot regeneration–related genes. In rice, overexpression of *WUS*, which promotes shoot regeneration rates, has been found to decrease grain yield [34]. Similarly, overexpression of *NAC DOMAIN CONTAINING PROTEIN 1*, which enhances root regeneration [35], has been shown to suppress plant growth in apple [36]. These adverse effects arise because the introduction of genes through the commonly used *Agrobacterium*-mediated transformation method results in the insertion of genes into the plant genome. Recent studies have shown that nanomaterial-based transient plant transformation is an efficient approach for delivering DNA and RNA without causing transgene integration. In tobacco (*Nicotiana benthamiana*), wheat, cotton, and arugula (*Eruca sativa*), carbon nanotubes enable efficient DNA delivery and protein expression without transgene integration [37]. Also, single-walled carbon nanotubes deliver small interference RNAs and show high silencing efficiency in intact plant cells [38]. These methods can be employed to deliver DNA or RNA that promotes shoot regeneration efficiency, and miR396a suppressors, such as STTM396a, are promising options due to their small size. Future research on the delivery of STTM396a into callus cells via carbon nanotubes for transient silencing of miR396a during shoot regeneration could enable the transgene-free enhancement of transgenic plant-generating efficiency.

Our results show that abundance of Sly-miR396a and pre-Sly-miR396a in SK were higher than that in SD (Fig. 3g and h). Analysis of transcription factor binding motifs in the promoter region of *Sly-MIR396A* revealed that several unique binding motifs for WRKY transcription factor present in SK (Fig. 3i and j). This suggests that WRKYs in SK may induce *Sly-MIR396A* expression, resulting in reduced shoot regeneration. Indeed, there are six *WRKY* genes in NG1 (*Solyc02g072190*, *Solyc01g079360*, *Solyc12g011200*, *Solyc07g055280*, *Solyc02g080890*, and *Solyc12g056750*). Among them, the Arabidopsis homolog of Solyc02g080890 is WRKY6, whose binding motif is exclusively present in the promoter of *Sly-MIR396A* in SK (Fig. 3j). Therefore, it is possible that the WRKY transcription factor encoded by *Solyc02g080890* may be responsible for the high expression of *Sly-MIR396A* in SK.

WRKY transcription factors are plant-specific proteins acting as either activators or repressors [39, 40]. Although WRKYs have been known to be related with abiotic and biotic stress responses, several members are involved in phytohormone-regulated plant development. For example, TaWRKY51 suppresses ethylene biosynthesis to improve lateral root development in wheat [41]. In addition, WRKY71 affects auxin homeostasis to accelerate shoot branching in Arabidopsis [42]. These reports support our hypothesis that WRKYs may be involved in shoot regeneration by affecting cytokinin-regulated Sly-miR396a abundance. Furthermore, expression of a number of *OsWRKY*s increases in response to cytokinin treatment in rice [43]. Based on the previous and our studies, it is possible that WRKYs are a key regulator to determine Sly-miR396a abundance, which is critical for shoot regeneration capacity in tomato.

## Materials and methods

### Plant materials and seed germination

The SD seeds were purchased from Koreon (cat. No: 02-05-99-023). The SK seeds were a gift from Dr Suk Yoon Kwon. The Gwangbok (cat. No: 02-0005-2014-92) and Minitomato (cat. No: 02-05-14-91) were purchased from Aram Seed. The Sinhonggwang seeds were purchased from Hyundai Seed (cat. No: 02-0005-2014-123). The tomato seeds from each genotype were sterilized by incubating them in 70% EtOH for 30 sec, followed by shacking them in a 1% NaOCl solution containing 0.01% Tween 20 for 7 min. Subsequently, the seeds were rinsed with sterile distilled water eight times and then dried on sterilized filter paper. After sterilization, the seeds were germinated on Murashige and Skoog (MS)–agar medium [4.33 g L^−1^ MS basal salt mixture (Duchefa, cat. No: M0222.0050), 3% sucrose, and 0.8% plant agar (Duchefa, cat. No: P1001.1000) with pH 5.8]. The plates were incubated in the dark for 4 days; then, they were transferred to long-day conditions (16-h light and 8-h dark) at 24°C.

### Shoot regeneration from tomato explants

To induce shoot regeneration, cotyledons or hypocotyls of 6-day-old seedlings were excised and incubated onto SIM (MS-agar medium containing 1.5 mg L^−1^ of either BA or zeatin) at 24°C under long-day conditions. Explants were transferred to the fresh SIM every 2 weeks. The regeneration frequency was determined by dividing the number of explants showing regenerated shoots by the total number of explants. Regenerated shoots taller than 1 cm were excised and transferred to MS-agar medium for 2 weeks to induce root organogenesis.

### RNA extraction and quantitative reverse-transcription PCR analysis

A portion of the explants located in proximity to the wound site and containing calli was excised for extracting total RNA using the RNeasy Plant Mini kit (Qiagen, cat. No: 74904). For quantitative reverse-transcription PCR analysis (qRT-PCR), 1 μg of total RNA was used to synthesize complementary DNA (cDNA) using TOPscript cDNA Synthesis kit (Enzynomics, cat. No: EZ005M). QRT-PCR was performed using TOPreal SYBR Green qPCR PreMIX (Enzynomics, cat. No: RT500M) in CFX Connect real-time PCR detection system (Bio-Rad, cat. No: 1855201). Primers used for qRT-PCR are listed in Supplementary Table S15. The *SlACTIN4* was used as a reference gene. To determine the abundance of Sly-miR396a, stem-loop qRT-PCR was performed [44]. Total RNA was extracted using the miRNeasy Mini kit (Qiagen, cat. No: 217004) and mixed with SL-miR396a oligomer (Supplementary Table S15). The mixture was incubated at 65°C for 5 min and then cooled on ice for annealing of the primer with miRNAs. The cDNA synthesis was performed using SuperScript IV reverse transcriptase (Thermo Fisher Scientific, cat. No: 18090010). The PCR was conducted using the Sly-miR396a forward and Universal reverse primers (Supplementary Table S15). For normalization, cDNA was synthesized using the TOPscript cDNA Synthesis kit (Enzynomics) with the same amount of total RNA used for stem-loop RT reactions. Subsequently, qRT-PCR was performed using the *SlACTIN4* (*Solyc04g011500*) primers.

### Vector construction

The *STTM396a* sequences [22] were inserted into the pH2GW7 vector using the gateway cloning method. For construction of the Cas9-gRNA vector targeting *SlPDS*, gRNA-coding sequences (GAGCTCGAGGTCGTCTTCTT) were inserted into the pHAtC vetor (Addgene, cat. No: 78098) using the *Aar*I restriction enzyme site.

### *Agrobacterium*-mediate transformation

The *STTM396a*-overexpressing (pH2GW7-STTM396a) vector was introduced into *A. tumefaciens* strain GV3101. *Agrobacterium* cells were cultured in YEP medium [10 g L^−1^ peptone (Gibco, cat. No: 211677), 10 g L^−1^ yeast extract (Gibco, cat. No: 211929), and 5-g L^−1^ NaCl (Samchun, cat. No: S0484)] until reaching OD_600_ = 0.5. The cells were then harvested by centrifugation and resuspended in 30 ml MS-liquid medium without sucrose. Cotyledon explants of the SK genotype were cocultivated in *Agrobacterium*-resuspended MS medium for 20 min with shaking every 5 min. Subsequently, the explants were placed onto sterile filter paper to remove excess liquid medium and incubated in the dark on SIM containing 200-μM acetosyringone for 2 days. The explants were then cultured on SIM containing 0.1-mg L^−1^ IBA, 500-mg L^−1^ cefotaxime, and appropriate selection markers under long-day conditions with a change of medium every 2 weeks until shoots were regenerated from the callus. For the transformation of mixed strains containing independent plasmids of pH2GW7-STTM396a or Cas9-gRNA, the concentration ratio was 1:1 during the cocultivation step.

### Analysis of gene editing

Genomic DNA was extracted from transformed T0 plants using HiYield Genomic DNA Mini kit (Real Biotech Corporation, cat. No: YGP100). To analyze insertion and deletion mutations in the *SlPDS* locus, we performed Sanger sequencing on the PCR-amplified target region. Sanger sequencing data were analyzed using ICE tool (https://ice.synthego.com).

### RNA-sequencing

Tomato explants incubated on SIM for 0, 6, 12, 18, 30, and 42 days were used for the analysis. Library construction was performed using 1 μg of total RNA by Illumina Stranded mRNA Prep kit (Illumina, cat. No: 20040534). Raw sequencing data were produced via the Illumina Hiseq X ten platform. The adaptor sequences and low-quality bases were trimmed, and the resulting clean reads were mapped to the *S. lycopersicum* reference genome sequence from the Sol Genomics Network database (ITAG4.0; https://solgenomics.net). Genes showing |log2 fold change| ≥ 1 and adjusted *P*-value <0.01 were annotated as DEGs. The R software was used to perform hierarchical clustering, utilizing the amap and gplots libraries. For small RNA-sequencing, the miRNeasy Mini kit (Qiagen) was used for the extraction of total RNA, including miRNAs, and TruSeq Small RNA Library Prep kit (Illumina, cat. No: RS-200) was used for the library construction. Only reads that were 15–30 nucleotides in length were included in the analysis. To determine miRNAs, the secondary structure of the mapped reads was predicted using the miR-PREFeR software [45]. Small RNAs that align to the genome and form a stem-loop structure were determined as miRNAs. Mature sequences and miRNA names are displayed in Supplementary Tables S7 and S8. The miRNA target genes were predicted using the miRNA target gene prediction software psRNATarget tool with default parameters [46]. The Biological Networks Gene Ontology tool was used for GO analysis, with a Benjamini–Hochberg-corrected *P* < 0.01. The network diagram displayed the significantly over-represented GO terms.

### Analysis of transcription factor binding motifs

Genomic locus of the Sly-MIR396a was searched in miRBase (https://mirbase.org). Promoters located ~1 kb from the *Sly-MIR396A* (NCBI reference sequence: NR_130090.1) were amplified using the pMIR396A_F and MIR396A_R primers (Supplementary Table S15), which span whole transcripts of precursor miR396a. Promoter sequences were identified using Sanger sequencing at Bioneer (Supplementary Fig. S3a). Transcription factor binding motifs were analyzed using the JASPAR database (https://jaspar.genereg.net) according to the instructions on the website. In detail, 1-kb upstream sequences from the *Sly-MIR396A* were analyzed using the Arabidopsis database of transcription factor binding motifs. Binding motifs showing a relative profile score more than 80% are selected and listed in Supplementary Tables S12 and S13. Unique binding motifs were analyzed by comparing matrix IDs using Microsoft Excel 2016.

### Statistical analysis

All statistical methods and the number of biological or technical replicates in each assay are described in the figure legends. Student’s *t*-test was performed using Microsoft Excel 2016. In box plots, the lower and upper quartiles bound each box, with the center line representing the median. The whiskers indicate the minimum and maximum values, and individual data points are shown in figures for each box plot. Raw data are represented in the source data files.

## Acknowledgements

We thank Dr. Suk Yoon Kwon for providing the tomato SK genotype seeds. This research was supported by the New Breeding Technologies Development Program (Project No. PJ01653001) provided by the Rural Development Administration of Korea, the Basic Research Program provided by the National Research Foundation of Korea (NRF-2023R1A2C1003963 and NRF-2022R1I1A2073565), and the KRIBB Research Initiative Programs (KGM5372322 and KGM1002311).

## Author contributions

S.-J.P., H.-S.K., and H.-J.L. conceived the study, designed the experiments, and analyzed the data. S.-J.P. performed tomato regeneration, transformation, and qRT-PCR with the assistance by J.H.Y. S.-J.P. and J.-S.P. prepared the plant materials and extracted total RNA for RNA- and small RNA-sequencing. S.-J.P. and H.-J.L. analyzed sequencing data. S.-J.P. and K.-B.M. analyzed gene editing. K.-B.M. constructed Cas9-gRNA vector targeting *SlPDS*. S.-J.P. and S.Y.S. prepared experimental reagents and plant materials. J.-H.J. and H.-S.K. provided experimental equipment and scientific discussions.

## Data availability statement

All data generated in this study are included in this article and the Source data files. The raw data for RNA- and small RNA-sequencing are deposited in KoNA (https://www.kobic.re.kr/kona). Accession numbers for RNA- and small RNA-sequencing data are KRA2301039 and KRA2301040, respectively.

## Conflict of interest 

None declared.

## Supplementary information

Supplementary data is available at *Horticulture Research* online.

## Supplementary Material

Web_Material_uhad291
