# Supplementary material for: MicroRNA396 negatively regulates shoot regeneration in tomato
Source: Hortic Res. 2024 Jan 2;11(2):uhad291. doi: 10.1093/hr/uhad291 (PMC10873581; doi:10.1093/hr/uhad291)
Supplement: Web_Material_uhad291 [file web_material_uhad291.zip › Supplementary Data Figures.pdf]

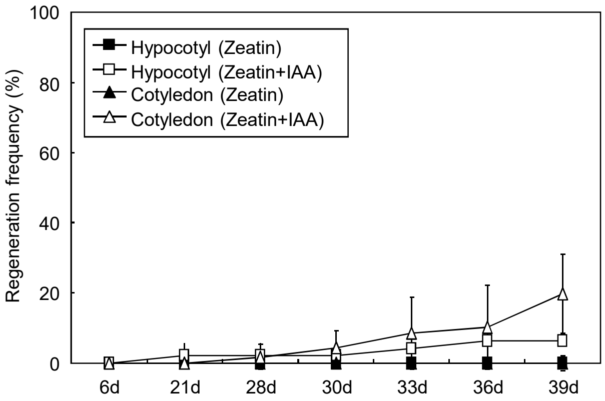

Supplementary Data Figure S1. Regeneration frequency of shoots in SK after auxin treatment. Hypocotyl and cotyledon explants from tomato seedlings grown for 6 days were incubated on SIM containing 1.5 mg L^{-1} zeatin or a combination of 1.5 mg L^{-1} zeatin and 0.1 mg L^{-1} indole-3-acetic acid (IAA).

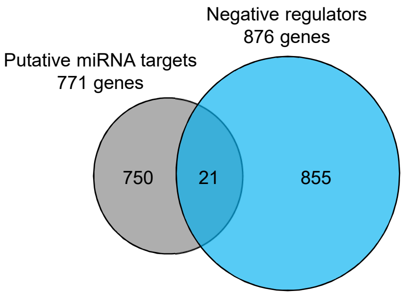

Supplementary Data Figure S2. Putative miRNA-target negative regulators of shoot regeneration. Venn diagram shows putative miRNA target genes found in Fig. 3b and putative negative regulators of shoot regeneration found in Fig. 2g.

(a)

```
pSly-MIR396A in SD
5′-ACATGCGAGAAATTGCTCAAATGATAAATATTCAAAAGTTAATAAGATAGAGAAAATATTAATATTCAAACGTTCATAAATATAAATGATATTAATCTTCTTAAACGGTCTTAA
AGAGTATACCATTCATTCACGAAAATTGCTCGAATGACAAATAGTTTTCTCTTGAAACACTAAAATTATCGGTTCAAGTTAAAAAAATACAAAAAATAAAAATATAAATTGACACTA
ATAAAAAGGAGGTAAGATTTTTTTATCTGATATATGTAATTTAAACAATTATATAACATTTTCTTCTGCTATTTTTGTTAGGCCACAACTAATATTTATTCCAGAAATATTTGCATA
ATGTAACTACCTCTTATAGTGGACCTGAGTGTGTAGATATTTATTTTTATAATATCAAACTTAAACTCTGTATTTTTTATGGTTCAATTTATGTAATGTATTTCTAAAAAAAATATA
TATATATATATATATATATATATTAACTTTTTTTTTTTACTATTAGGCATAAAATTTATAAATATTAGTCCTCAAAATTTAACTTTTGAAATTACATCAAATATATTATTATTGCTA
GAAGAATTCAAAATTATTTTTACACATAACTAAATAATTTGTAACCACATATTTATTTAAGTCTTGTTCTAAATAACAAGTTTCAAAAATTTATTTATTTTTATTTAATAATAGTGC
CACTTAAATTAGAAAAAAACCCAATATATTTATTCAAAAAGCAGGTTTGTTCAGAGTTTTGTTATAGACCCAATCCAATATTCTTCTTGGGATCAGCAGTAAAATGATACCCACCCT
TACTCCACCCCACCCCAACCTACCCACTCAAATTACCCCTATAAATAATGTCTAGTCTTCTCCTCTCTCAACACTTCACTTCTTCTAAAATTCTCATCCAGTGAAAACAATTTTTTT
TCCCTCTAGGAATCTCAAAAAACCATATCTAGGTTTAATGAGATTCTATTTATAGAGATTTTTGTGTACAAAA-3′

pSly-MIR396A in SK
5′-ACATGCGAGAAATTGCTCAAATGATAAATATTAAAAAGTTAATAAGATAGAGAAAATATTAATATTCAAACGTTCATAAATATAAATGATATTAATCTTCTTAAACGGTCTTAA
AGAGTATACCATTCATTCACGAAAATTGCTCGAATGACAAATAGTTTTCTCTTGAAACACTAAAATTATCGGTTCAAGTTAAAAAAATACAAAAAATAAAAATAAAAATTGACACTA
ATAAAAAGGAGGTAAGATTTTTTTATCTGATATATGTAATTTAAACAATTATATAACATTTTCTTCTGCTATTTTTGTTAGGCCACAACTAATATTTATTCCAGAAATATTTGCATA
ATGTAACTACCTCTTATAGTGGACCTGAGTGTGTAGATATTTATTTTTATAATATCAAACTTAAACTCTGTATTTTTTATGGTTCAATTTATGTAATGTATTTCTAAAAATATATAT
ATATATATATATAAACTTTTTTTTTTTACTATTAGGCATAAAATTTATGAATATTAGTCCTCAAAATTCAACTTTTGAAATTACATCAAATATATTATTATTGCTAGAAGAATTCAAA
ATTCTTTTTACACATAACTAAATAATTTGTAACCACATATTTATTTGAGTCTTGTTCTAAATAACAAGTTTCAAAAATTTATTTATTTTTATTTAATAATAGTGCCACTTAAATTAG
AGAAAAACCCAATATATTTATTCAAAAAGCAGGTTTGTTCAGAGTTTTGTTATAGACCCAATCCAATATTCTTCTTGGGATCAGCAGTAAAATGATACCCAGCCTTACTCCACCCCA
CCCCAACCTACCCACTCAAATTACCCCTATAAATAATGTCCAGTCTTCTCTTCTCTCAACACTTCACTTCTTCTAAAATTCTCATCCAGTGAAAACAATTTTTTTTTCCTCTAGGAA
TCTCAAAAAATCATATCTAGGTTTAATGAGATTCTATTTATAGAGATTTTTGTGTACAAAA-3′
```

(b)

```
SD 201 ATACAAAAAATAAAAATATAAATTGACACTAATAAAAAGGAGGTAAGATTTTTTTATCTGATATATGTAATTTAAACAATTATATAACATTTTCTTCTGC 300
                         X
SK 201 ATACAAAAAATAAAAATAAAAATTGACACTAATAAAAAGGAGGTAAGATTTTTTTATCTGATATATGTAATTTAAACAATTATATAACATTTTCTTCTGC 300
                210       220       230       240       250       260       270       280       290       300
                             #1

SD 301 TATTTTTGTTAGGCCACAACTAATATTTATTCCAGAAATATTTGCATAATGTAACTACCTCTTATAGTGGACCTGAGTGTGTAGATATTTATTTTTATAA 400

SK 301 TATTTTTGTTAGGCCACAACTAATATTTATTCCAGAAATATTTGCATAATGTAACTACCTCTTATAGTGGACCTGAGTGTGTAGATATTTATTTTTATAA 400
                310       320       330       340       350       360       370       380       390       400

SD 401 TATCAAACTTAAACTCTGTATTTTTTATGGTTCAATTTATGTAATGTATTTCTAAAAAAAATATATATATATATATATATATATATATTAACTTTTTTTT 500
                                                                                                    X
SK 401 TATCAAACTTAAACTCTGTATTTTTTATGGTTCAATTTATGTAATGTATTTCTAAAA-----------ATATATATATATATATATATAAAC-TTTTTTT 488
                410       420       430       440       450                 460       470       480

SD 501 TTTACTATTAGGCATAAAATTTATAAATATTAGTCCTCAAAATTTAACTTTTGAAATTACATCAAATATATTATTATTGCTAGAAGAATTCAAAATTATT 600
                                X                   X                                                      X
SK 489 TTTACTATTAGGCATAAAATTTATGAATATTAGTCCTCAAAATTCAACTTTTGAAATTACATCAAATATATTATTATTGCTAGAAGAATTCAAAATTCTT 588
       490       500       510       520       530       540       550       560       570       580
                                             #2 to #11
```

Supplementary Data Figure S3. Analysis of different transcription factor binding motifs in promoters of *Sly-MIR396A* between SD and SK.

a Promoter sequences located approximately 1 kb upstream from the *Sly-MIR396A* in SD and SK. Promoters were amplified by PCR and were subjected to Sanger sequencing.

b Location of different transcription factor binding motifs between SD and SK. Alignment of promoter sequences of *Sly-MIR396A* in SD and SK was performed using the SnapGene software. Different transcription factor binding motifs were underlined. Numbers indicate types of transcription factor binding motifs in Fig. 3j.

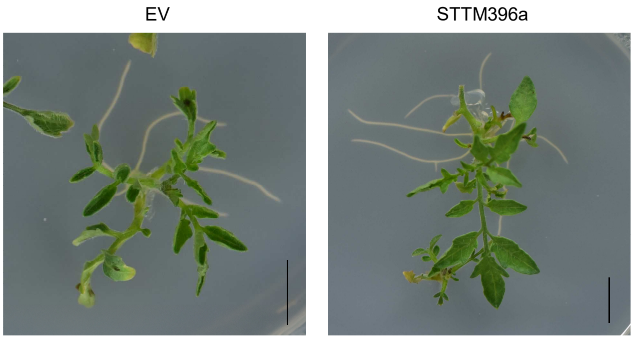

Supplementary Data Figure S4. Representative images of rooting transgenic plants. Regenerated shoots harboring EV or STTM396a were excised and cultured in MS-agar medium for 2 weeks. Scale bars, 1 cm

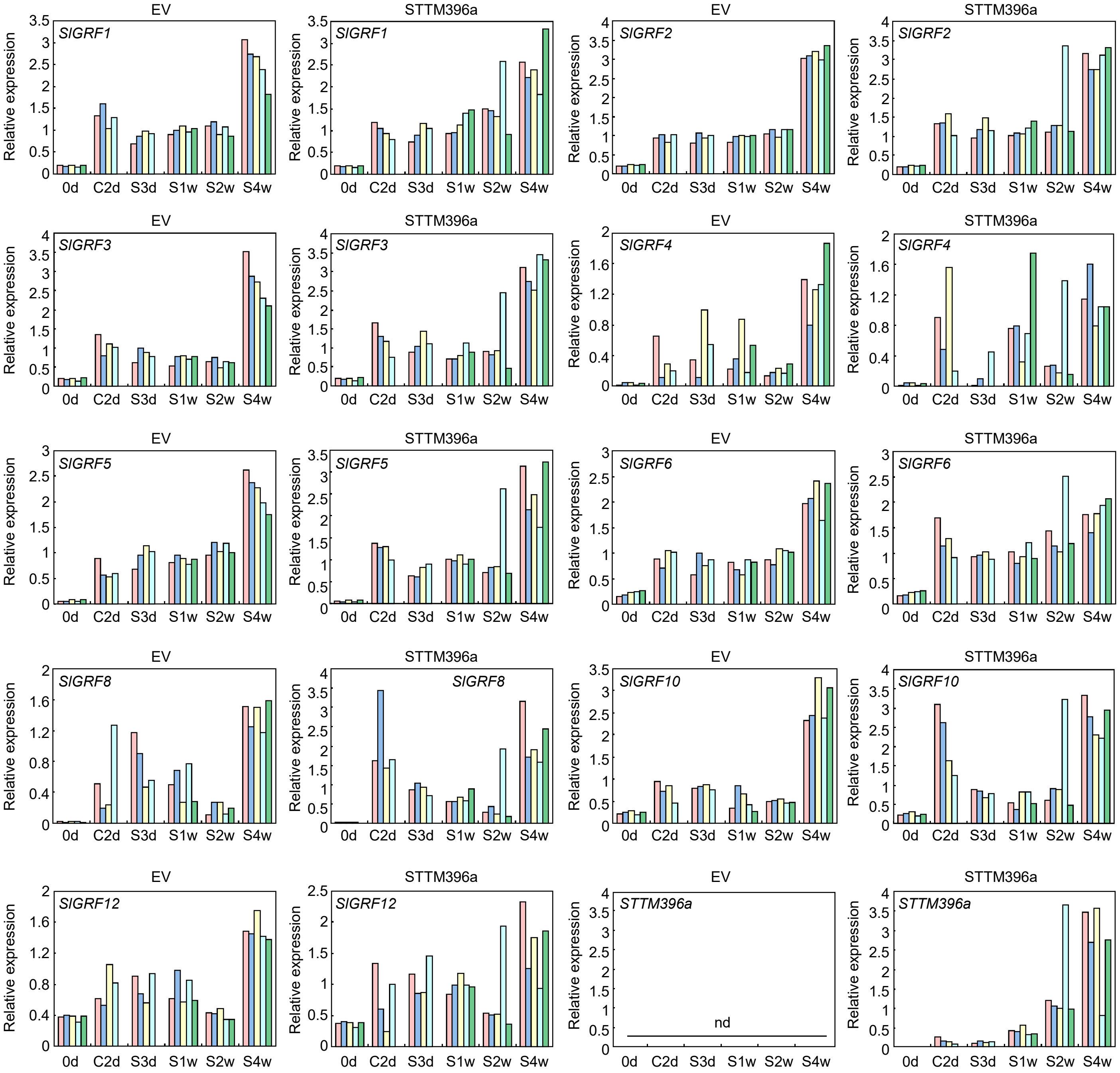

Supplementary Data Figure S5. Expression of *9 SlGRFs* and *STTM396a* after transformation of empty vector (EV) and *STTM396a*-overexpression vector (STTM396a) into SK cotyledon explants. Transformed T0 explants were harvested for total RNA extraction. These data represent relative expression for each replicate shown in Fig. 4d. C, co-cultivation medium; S, shoot-inducing medium; d, days after culture; w, weeks after culture; nd, not detected.
